# APOA5 Q97X Mutation Identified through homozygosity mapping causes severe hypertriglyceridemia in a Chilean consanguineous family

**DOI:** 10.1186/1471-2350-13-106

**Published:** 2012-11-15

**Authors:** Catalina Dussaillant, Valentina Serrano, Alberto Maiz, Susana Eyheramendy, Luis Rodrigo Cataldo, Matías Chavez, Susan V Smalley, Marcela Fuentes, Attilio Rigotti, Lorena Rubio, Carlos F Lagos, José Alfredo Martinez, José Luis Santos

**Affiliations:** 1 Department of Nutrition, Diabetes and Metabolism, School of Medicine, Pontificia Universidad Católica de Chile, Alameda 340, Santiago, Chile; 2 Department of Statistics, School of Mathematics, Pontificia Universidad Católica de Chile, Santiago, Chile; 3 Department of Pharmacy, School of Chemistry, Pontifica Universidad Católica de Chile, Santiago, Chile; 4 Department of Nutrition and Food Sciences, Physiology and Toxicology, University of Navarra, Pamplona, Spain

**Keywords:** Hypertriglyceridemia, Genetic, Chylomicronemia, Mutation, Homozygosity mapping, APOA5

## Abstract

**Background:**

Severe hypertriglyceridemia (HTG) has been linked to defects in LPL, APOC2, APOA5, LMF1 and GBIHBP1 genes. However, a number of severe HTG cases are probably caused by as yet unidentified mutations. Very high triglyceride plasma levels (>112 mmol/L at diagnosis) were found in two sisters of a Chilean consanguineous family, which is strongly suggestive of a recessive highly penetrant mutation. The aim of this study was to determine the genetic locus responsible for the severe HTG in this family.

**Methods:**

We carried out a genome-wide linkage study with nearly 300,000 biallelic markers (Illumina Human CytoSNP-12 panel). Using the homozygosity mapping strategy, we searched for chromosome regions with excess of homozygous genotypes in the affected cases compared to non-affected relatives.

**Results:**

A large homozygous segment was found in the long arm of chromosome 11, with more than 2,500 consecutive homozygous SNP shared by the proband with her affected sister, and containing the APOA5/A4/C3/A1 cluster. Direct sequencing of the APOA5 gene revealed a known homozygous nonsense Q97X mutation (p.Gln97Ter) found in both affected sisters but not in non-affected relatives nor in a sample of unrelated controls.

**Conclusion:**

The Q97X mutation of the APOA5 gene in homozygous status is responsible for the severe hypertriglyceridemia in this family. We have shown that homozygosity mapping correctly pinpointed the genomic region containing the gene responsible for severe hypertriglyceridemia in this consanguineous Chilean family.

## Background

Hypertriglyceridemia (HTG) is defined as an elevation of plasma triglycerides (TG) above 1.7 mmol/L, and considered as severe HTG when it reaches plasma levels above 5.6 mmol/L [1]. HTG is a common dyslipidemia in the general population, with a estimated prevalence in Chile of about 30% [2]. HTG can be divided into primary and secondary types. Primary HTG is usually caused by a single genetic defect, while secondary HTG is multifactorial and associated with health conditions such as obesity, diabetes mellitus, insulin resistance or certain drugs [3].

Following the Fredrickson classification, primary HTG such as hyperlipidemia (HLP) type IV or type V, are characterized by the cumulative effect of several relatively common genetic variants in association with adverse environmental factors [4,5] On the other hand, monogenic HTG usually manifests clinically as HLP type I. The main genes known to be responsible for this disorder are LPL and APOC2 [6]. In recent years, other genes causing severe HTG have been identified such as APOA5, GPIHBP1 and LMF1 [7-15]. However, it is estimated that the metabolic and genetic defect underlying primary HTG is only known in few cases, indicating that there are probably other unknown genes involved in the development of this disorder [4,16]. On the other hand, Genome Wide Association Studies (GWAS) have identified gene variation related to discrete variations in plasma TG levels in the general population [4]. Thus, common variants in genes such as GCKR, TRIB1, MLXIPL, GALNT2, APOB, APOA5, APOE, LPL, APOC2, APOC3, ANGPTL3 and NCAN (for acronyms see Additional file 1: Table S1) have shown a significant, although modest effect on plasma TG [4,5,17-21]. Genotype-phenotype associations from GWAS and family studies indicated that common and rare variants in candidate genes are related to mild and severe HTG [16,20-22]. On the other hand, homozygosity mapping is a straightforward approach based on linkage analysis suitable for the study of autosomal recessive diseases in consanguineous families [23]. This strategy has been successfully employed in the identification of the genetic cause of diseases such as congenital generalized lipodystrophy, Allstrom syndrome or complete achromatopsia, among others [24-26]. Thus, this approach may be used for the identification of causative genes of severe HTG in consanguineous families with multiple family members affected.

The aim of the present study is to identify the gene and mutation responsible for the severe HTG (plasma TG >112 mmol/L) found in two sisters of a consanguineous Chilean family. For this purpose, we performed a genome scan using nearly 300,000 biallelic markers (Single Nucleotide Polymorphism: SNP). Through extensive search of regions showing consecutive homozygous markers (homozygosity mapping), we found a large segment of homozygosity in chromosome 11q23 comprising about 2,896 SNP shared by two affected sisters and non-shared by the unaffected siblings. This region includes the APOA5/A4/C3/A1 gene cluster, which in turn contains APOA5 as a primary candidate gene. Direct sequencing of the APOA5 gene revealed a homozygous nonsense mutation (p.Gln97Ter; Q97X) in both affected sisters. In this study, we show that homozygosity mapping is a useful and efficient strategy to identify genetic loci responsible for dyslipidemias in consanguineous families.

## Subjects and methods

### Subjects

**Figure 1:**
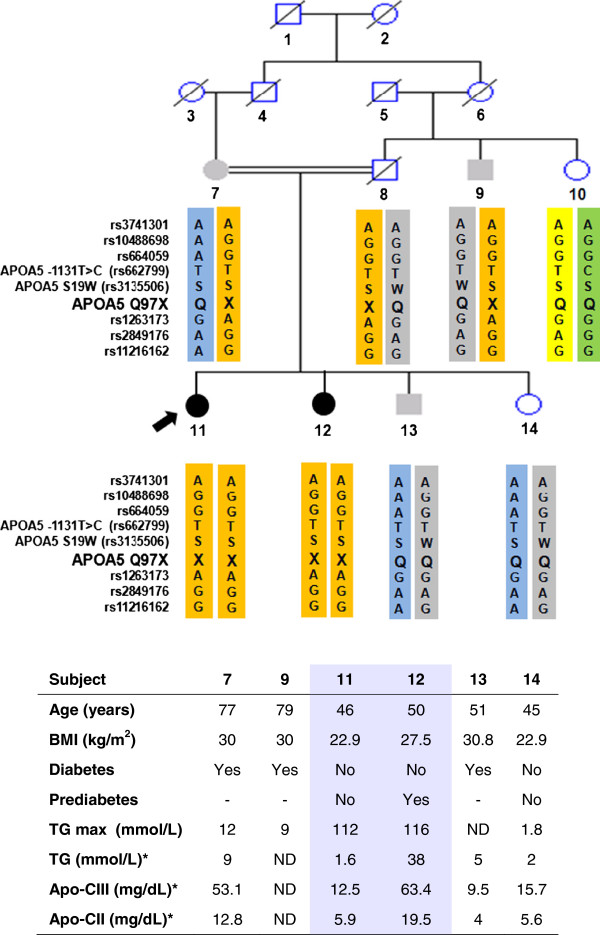
**Clinical characteristics and haplotypes of the APOA5 gene and its flanking region in a consanguineous Chilean family with hypertriglyceridemia.** Arrow: proband (subject 11); Solid symbols: patients with triglycerides (TG) >56 mmol/L; Grey-filled symbols: patients with 5.6 < TG < 22 mmol/L; Double line: consanguineous marriage. Rs numbers represents SNPs flanking the APOA5 gene. In table: Grey-filled columns: patients with severe chylomicronemia (TG >56 mmol/L), homozygous for 97X mutation; * Tests taken on 2006; TG: Triglycerides; BMI: Body Mass Index; ND: Not Determined; Plasma Apo C-III normal range: 1.2 – 17.2 mg/dL. * Subject`s 8 haplotype was inferred from relative’s haplotypes.

Figure 1 shows the genealogy of the most relevant subset of the consanguineous family under study (see Additional file 1: Figure S1 for the complete pedigree). Saliva samples were obtained for DNA analysis from 19 family members using the Oragene DNA OG-250 kits ( http://www.dnagenotek.com). We conducted a brief medical interview with all family members, requesting their medical history, past surgeries and diseases and medical treatments. We further evaluated lifestyle variables and anthropometry. Written informed consent was obtained from the proband and her relatives. Written informed consent was obtained from the patient for publication of this Case report and any accompanying images. A copy of the written consent is available for review by the Editor of this journal. The study protocol was approved by the Ethics Committee of the School of Medicine of the Pontificia Universidad Católica de Chile (protocol number 09–112).

The proband (subject 11) is a 46-year-old woman who, since age 22, has presented severe HTG, with maximum plasma TG levels of 112 mmol/L. After suffering acute pancreatitis during her first pregnancy, she required weekly plasmapheresis during subsequent pregnancies in order to maintain plasma TG levels below 11 mmol/L. The proband has no co-morbidities associated with HTG and shows normal body weight. Currently, her condition is kept under control with diet and associated therapy (fibrates and nicotinic acid). Her sister (subject 12), age 50, has presented severe HTG since the age of 30. She has reached a maximum TG level of 116 mmol/L and has suffered several episodes of acute pancreatitis. She has been diagnosed as pre-diabetic, is overweight, and has required emergency plasmapheresis on several occasions. Currently, she is under treatment with fibrates and omega-3 fatty acids.

The brother of the proband (subject 13) has an intermediate phenotype, presenting TG levels >5.6 mmol/L, but not higher than 22 mmol/L. He has not presented episodes of acute pancreatitis. He is diabetic and obese. Although he has previously required fibrates, his condition is currently kept under good control with atorvastatin only. Another younger sister of the proband (subject 14) has never presented severe HTG. The father (subject 8) was diagnosed with diabetes and died at age 69 of coronary heart disease. Subject 9, a paternal uncle of the affected sisters, is diabetic, overweight and presents an intermediate phenotype with a maximum known level of TG of 9 mmol/L. The mother of the affected sisters (subject 7) is diabetic, obese, and also shows an intermediate phenotype with a maximum TG of 12 mmol/L, and without previous episodes of acute pancreatitis.

In 2006, levels of apolipoprotein C-II and C-III, lipid profile and blood glucose levels were measured in most of the members of the family (see lower part of Figure 1). It is worth mentioning that the mother and the sister of the proband showed elevated levels of Apo C-II and Apo C-III.

### Genome scan

Genomic DNA from saliva was checked for integrity, treated with RNAse and fluorometrically quantified (Qubit platform, Invitrogen). A genome-wide scan for linkage was performed using the Human Cyto SNP-12 panel ( http://www.illumina.com), which determines approximately 300,000 SNP with an average distance of 9.7 kb (see panel features in Additional file 1: Table S2). Genotyping was carried out at the Spanish National Genotyping Centre (CeGen; http://www.cegen.org). Using our own controls, the genotyping success rate, reproducibility (two sets of duplicate samples) and Mendelian consistency (case-parent trios from the family) were above 99%.

### Statistical analysis

Genotypes were analyzed with the PLINK software. R routines were created at the School of Mathematics of the Pontificia Universidad Católica of Chile to display areas of homozygosity (routines are available under request). Using such R routines, genome regions showing excess homozygosity (initially defined in an arbitrary way as >100 consecutive SNPs) shared by the two affected sisters, but not by healthy siblings, were identified as candidate regions for containing a disease locus responsible for the severe HTG.

We also carried out a formal LOD score calculation to assess linkage in the regions that showed excess homozigosity [23]. Genetic markers flanking the APOA5/A4/C3/A1 cluster were assessed first since this locus showed the largest number of consecutive homozygous SNPs in the initial scan. To reduce the computational burden of multipoint linkage analysis and to increase marker information, the complete haplotype, defined by six SNPs flanking the APOA5/A4/C3/A1 cluster (rs3741301, rs10488698 rs6640, rs1263173, rs2849176 and rs11216162), was used for LOD score calculations under a recessive model of complete penetrance. We used a modified version of the MLINK 4.03 program compiled for analysis of pedigrees with multiple consanguinity-loops [25]. A LOD score > 3 is highly suggestive of linkage, while LOD scores < −2 suggest linkage exclusion [25]. (See Additional file 1: Methods for specification of parameters used in the linkage analysis).

### Genetic analysis of APOA5 gene

Primers were designed for sequencing APOA5 exons and intron-exon boundaries. Genomic DNA was amplified through PCR (see detailed information in Additional file 1: Methods). Once the causative mutation was detected (Q97X), a PCR-RFLP assay was developed to identify such mutation in other family members and to verify its absence in 50 anonymous DNA samples. Additionally, S19W (rs3135506) and -1131T>C (rs662799) polymorphisms of the APOA5 gene were genotyped in all family members [9] (detailed information in Additional file 1: Methods).

## Results

**Figure 2:**
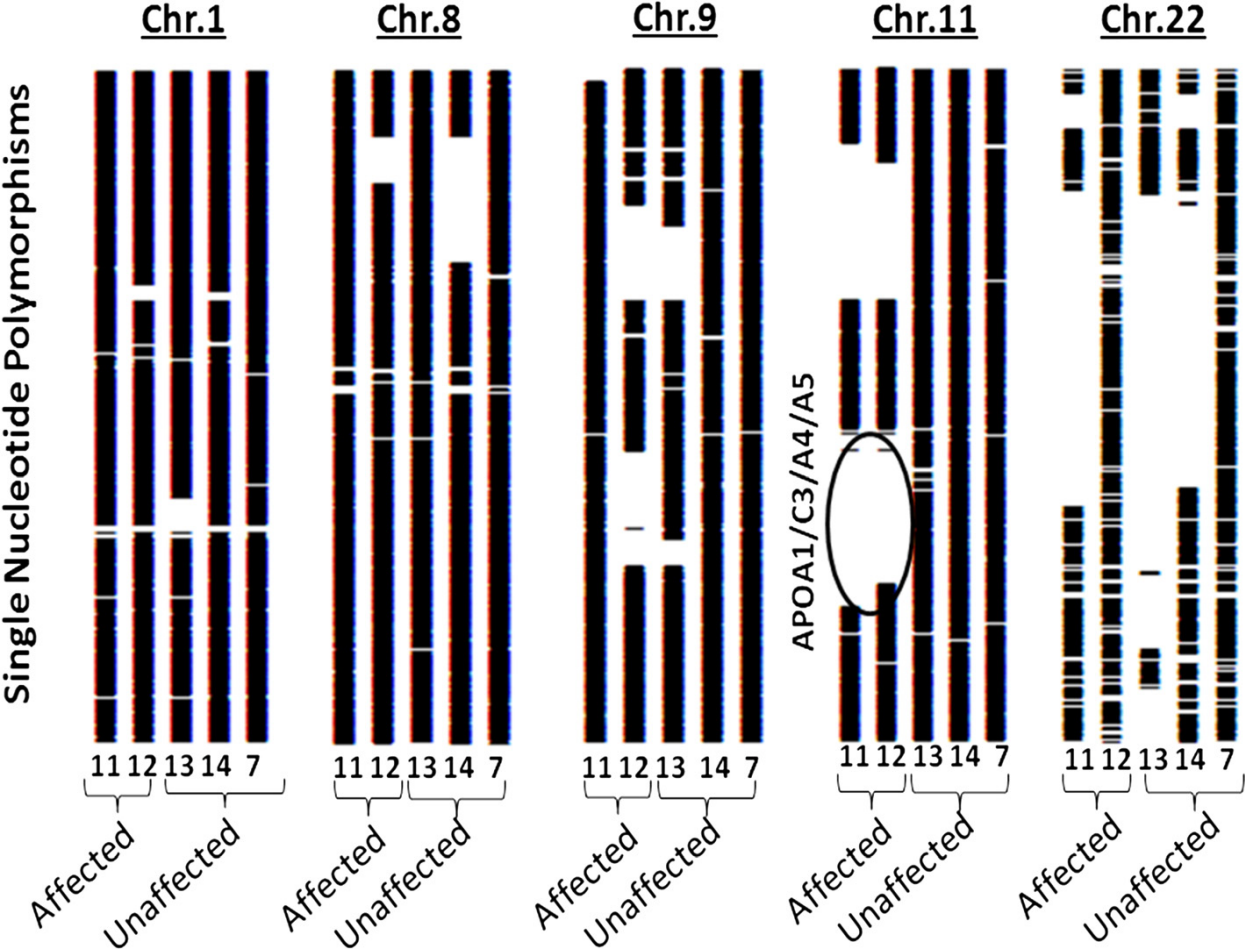
**Homozygous and heterozygous regions determined through homozygosity mapping in a consanguineous Chilean family with hypertriglyceridemia.** Chr: chromosome; Numbers, 11: proband; 12: affected sister; 13 and 14: nonaffected siblings; 7: mother; Solid regions: heterozygosity in markers; Open regions: homozygosity in markers.

Fifteen regions were identified as a) having excess of homozygosity (arbitrarily defined as > 100 consecutive homozygous SNP) shared by the two affected cases (subjects 11 and 12), and simultaneously, b) showing a substantial heterozygosity in the non-affected sister (subject 14; with heterozygosity found in at least 20% of the SNP). The size of these regions ranged from 13,310 kb to 195,339 kb, (from 100 to 3,000 consecutive homozygous SNP) (Additional file 1: Table S3). The APOA5/A4/C3/A1 cluster is located in the largest homozygous region shared by the two affected cases (2,860 consecutive homozygous SNP) (Figure 2). Given that the APOA5/A4/C3/A1 cluster has been described in the literature as responsible for increased plasma TG levels, this locus was considered as the primary candidate region responsible for severe HTG in the family [27]. The possible location of a causal mutation in this locus was furthermore supported by classical linkage analysis under a recessive model of complete penetrance, with a calculated LOD score of 2.02.

Considering the clinical characteristics displayed by the affected cases in the family under study (subjects 11 and 12; HLP type V), the most probable candidate gene within the APOA5/A4/C3/A1 cluster for carrying a mutation was the APOA5 gene [9,28]. Therefore, we proceeded to sequence analysis this gene, which led to the detection of a known single nucleotide nonsense substitution in exon 4, which changes a glutamine at position 97 into a premature stop codon (mutation NM_001166598.1:c.289C>T; NP_001160070.1:p.Gln97Ter, named from now on Q97X) (Additional file 1: Figure S3). Sequencing and PCR-RFLP analysis showed that the proband and her affected sister (subjects 11 and 12) are homozygous for this nonsense mutation, while subjects 7, 9 and 16 are heterozygous for the mutation. Additionally, subjects 13 and 14 were found to be non-carriers (Figure 1; Additional file 1: Figure S1 and S3). Descendants of subjects 11 and 12 are obligatory heterozygous carriers, although their DNA was not available for this study (Additional file 1: Figure S1). The mutation Q97X was not found in other family members among those with available DNA, being absent in 50 anonymous DNA samples from Chilean unrelated blood donors.

We also evaluated the common genetic variants APOA5 S19W and -1131T>C in this family as they have been significantly related to multifactorial hypertriglyceridemia [28,29] (Figure 1 and Additional file 1: Figure S3). Heterozygous carriers of the Q97X mutation showed homozygous wild-type genotypes for APOA5 S19W and -1131T>C, except for subject 8 (inferred genotype) and subject 9, who showed a heterozygous genotype in the APOA5 S19W polymorphism.

## Discussion

We have conducted a genome-wide search for shared homozygosity in affected sibs in order to identify the mutation responsible for the severe HTG in a consanguineous Chilean family. There are three strategies commonly used to search for rare causative mutations in severe monogenic diseases in families: a) genome or exome sequencing, b) linkage analysis followed by sequencing of candidate genes within the genomic area significant for linkage, and c) resequencing all candidate genes related to the disease. Exome/genome sequencing (a) is becoming with no doubt a key tool to find disease-causing mutations [30]. The use of exome sequencing approach would have been clearly successful in the identification of the genetic cause of HTG in our family. However, in a more general context, there are still some challenges associated with the exome sequencing strategy. It has been estimated that each individual exome harbors around 20,000 sequence variants in comparison with the human reference genome, including 5,000 variants affecting amino acid sequence. After filtering with common genetic variation found in databases, still many variants are left as candidate causal, even if we consider recessive defects [31,32]. Linkage analysis (b) is a classical genetic strategy to search for causal loci responsible of genetic diseases which is based on the use of a map of genetic markers (microsatellite markers or SNP) across the genome. A special case of linkage analysis is the so-called homozygosity mapping strategy, which has been extensively used in the study of genetic causes underlying diseases with recessive effect in consanguineous families. In these types of studies, the objective is to identify homozygous markers shared by the affected cases that pinpoint genomic regions that may contain the responsible gene and mutation [23]. It is important to mention that the combination of genome/exome sequencing and homozygosity mapping is an appealing approach that has been successfully applied in the identification of mendelian diseases [33]. Direct sequencing of candidate genes (c) is conceptually the most straightforward approach to find causal mutations. However, there are some important considerations that should be taken into account regarding the laboratory effort required for resequencing studies. Sequencing the five most plausible candidate genes for severe HTG (LPL, APOC2, APOA5, LMF1 and GPIHBP1) would include the analysis of ten exons of the LPL gene (main candidate gene), four exons of the APOA5 gene, thirteen exons of the LMF1 gene, four exons of the GPIHBP1 gene and five exons of the APOC2 gene (a total of 36 exons). If no mutation is found initially in these genes, next logical steps would be the search for novel mutations in genes with common variants that have been associated with multifactorial HTG through genome-wide association studies such as for example GCKR (19 exons), and possibly testing genes with loss-of-function mutations causing hypotriglyceridemia (for example, 6 exons of the ANGPTL3, 29 exons of the APOB gene, among others). Therefore, resequencing genes with traditional techniques may represent an important investment in terms of time, especially if the causative mutation is not found in the initial steps. In contrast, the timeframe we have employed in this homozygosity mapping study in submitting DNA to genome-wide analysis, locating the causative gene and discovering the mutation was of approximately 6 months. This was possible because the homozygosity mapping directly led us to the chromosomal region in which the causal mutation is located. As stated before, another advantage of the homozygosity mapping strategy is that limits the search for candidate mutations provided by exome sequencing in consanguineous families [34], and provides the possibility to identify new genes not previously described as responsible for extreme phenotypes. On the contrary, the main limitation of homozygosity mapping is that it requires consanguineous families. It is also important to notice that sometimes (especially in small families), this method pinpoints a very large chromosomal region, in which hundreds of genes could be responsible of the causal mutation. It is also important to mention that diseases caused by compound heterozygous are not detectable using this strategy. In our study, homozygosity mapping correctly pinpointed the region carrying the gene responsible for the disease [9,28] in 11q23 that contains APOA5/A4/C3/A1 gene cluster. After Sanger sequencing, the Q97X mutation of the APOA5 gene was identified in a homozygous state in the two affected sisters and in a heterozygous state in other family members. Although statistical power for finding significant LOD scores is always increased with larger number of affected and unaffected relatives, it is noteworthy to mention that the discovery of mutations is possible using only a single family [25]. Given the arguments presented above, we believe that homozygosity mapping is a valid and fast approach to locate causal recessive mutations in consanguineous families. This power of this strategy is enhanced when used in combination with resequencing of candidate genes or exome sequencing techniques.

The APOA5 gene is located on chromosome 11q23, within the APOA1/C3/A4/A5 gene cluster, a well-studied region that had previously been associated with variations in plasma TG [27]. Apo A-V protein was discovered in 2001 by two research teams simultaneously [35,36]. This apolipoprotein is synthesized in the liver and is associated with very low density lipoprotein (VLDL), chylomicrons (QM) and high-density lipoprotein (HDL). Its concentration in plasma is very low (0.1-0.4 μg/ml), which is approximately 300-fold lower than Apo C-III and roughly 2000-fold lower than Apo A-I [9,28,37]. It has been proposed that Apo A-V influences plasma TG levels primarily by increasing the catabolism of TG-rich lipoproteins through stimulation of LPL [38-41] or alternatively by inhibiting the rate of production of VLDL [42]. It was demonstrated that adenoviral overexpression of Apo A-V in mice is related to a 70% reduction in TG levels compared to wild-type mice, while Apoa5 knock-out mice showed a four-fold plasma levels relative to controls [43,44]. In humans, an inverse association between plasma Apo A-V and TG levels has been reported in cases of homozygous APOA5 nonsense mutations [9], whereas other reports indicate higher ApoA-V plasma concentrations in HTG patients [45]. Interestingly, it has been shown that intravenous injection of ApoA-V into apoa5 knock-out mice induces a 60% decrease in plasma TG after four hours [46].

An excess of rare APOA5 variants have been found in HTG patients, providing evidence for the importance of APOA5 in this disorder [47]. Mutations in the APOA5 gene have been described in cases of severe chylomicronemia, such as the Q148X mutation, Q139X mutation, IVS3+3G>C and three heterozygous missense variants [48-51]. The phenotypic expression of these mutations is highly variable, especially in heterozygous subjects, who usually require additional adverse environmental triggers (e.g. obesity, diabetes) or the presence of other susceptibility genetic variants. The Q97X mutation found in this study was described for the first time by Oliva et al. [52] and subsequently by Charriere et al. [53]. After the removal of the endoplasmic reticulum signal peptide of 23 amino acids, this mutation would cause truncation at residue 74 of the mature protein (343 amino acids) [54], creating a predicted 10 kDa peptide instead of the 39 kDa complete protein. This peptide, if synthesized (see discussion on NMD below), would lack the essential domain for lipid binding and LPL activation (residues 192 to 238) and therefore is expected to be non-functional [41,55] (Additional file 1: Figure S4 shows our own prediction model of the Apo A-V protein structure). In these previous reports on homozygous patients carrying the Q97X mutation, neither wild-type nor truncated Apo A-V were detected in plasma, thus suggesting a complete Apo A-V deficiency [52,53].

There is a remarkable variability shown by affected HTG cases that are carriers of the Q97X mutation in different studies, especially in terms of drug requirements, responsiveness to treatment, age of onset, TG plasma levels and other clinical manifestations [52,53] (See Additional file 1: Table S4). For example, heterozygous Q97X carriers reported by Oliva et al. showed phenotypes ranging from severe hyperchylomicronemia to normotriglyceridemia [52]. It is possible that partial Apo A-V deficiency by itself is not sufficient to trigger severe HTG unless adverse environmental stimuli overload the lipolytic system [52,53]. Similar observations have been reported for HTG cases with heterozygous genotypes in other APOA5 mutations (Q139X, Q148X) [9,48,49,56]. In our study, two heterozygous carriers of the mutation (subjects 7 and 9) are both diabetic and overweight, showing an intermediate phenotype of HTG. They developed severe HTG although never reached TG values as high as the two homozygous 97X sisters, with no history of acute pancreatitis. On the other hand, the two homozygous affected sisters showed triglycerides levels much higher than in the cases previously described (112 mmol/L compared to 11 mmol/L in the case reported by Oliva and 40 mmol/L in the case reported by Charriere) [52,53]. It is possible that our patients carry other alleles that contribute to this more severe phenotype, such as common or rare gene variants described in the APOE, LPL or APOB genes [16,21,22].

The fact that heterozygous carriers of this autosomic recessive disease also show some pathologic manifestations indicates either haploinsufficiency or a dominant negative effect of the truncated protein. In the first case, LPL may play its role properly in adequate conditions maintaining plasma TG levels within a normal range. However, if the system is subjected to an overload due to adverse environmental factors, the enzyme is no longer able to maintain normal TG metabolism, and HTG appears. In the second case, it can be postulated that the truncated peptide has a negative effect by itself. In this context, NMD (Nonsense Mediated mRNA Decay) is a cellular mechanism that eliminates mRNA that carries a premature stop codon in order to prevent the synthesis of truncated proteins. It has been proposed that NMD is not effective on premature stop codons located in the last exon of the gene or less than 50 nucleotides upstream of the last splice junction [57]. Thus, it is possible that the NMD mechanism does not occur in the Q97X premature stop codon, and the truncated protein is synthesized and itself interferes with the mature Apo A-V. Although previous reports of Q97X mutations did not find the truncated peptide plasma [52,53], one report indicates that the mature protein did not properly bind to TG-rich lipoproteins in Q97X heterozygotes [53], suggesting that truncated peptide may be synthesized but not secreted to the plasma.

APOA5 is a relatively polymorphic gene showing common variants in the promoter region (−1131T>C) and in the signal peptide (S19W) that have been previously associated with elevated plasma TG levels [28,29,58]. Recently, APOA5 -1131T>C polymorphism has been associated with higher plasma TG levels in a large epidemiologic study (16% increase for every C allele inherited) [59]. The presence of susceptibility alleles for HTG in APOA5 -1131T>C and S19W might be important in heterozygous carriers of Q97X when the mutation is located in a different haplotype than the susceptibility common variants APOA5 -1131C and 19W. In the French pedigree described by Charriere et al., most of the Q97X heterozygotes carried another susceptibility haplotype on the second chromosome [52]. Similar results were observed in the report by Marcais et al. [49]. In our family, only one of the three Q97X heterozygous subjects showed the 19W susceptibility allelic variant (subject 9), while the other heterozygous subjects showed the wild-type genotype in the -1131T>C variant (Figure 1). It is remarkable that even though subject 9 also carries the 19W susceptibility variant, he has not developed a more severe disease compared with heterozygous subject 7, who shows wild type S19 homozygous genotype. Nevertheless, we consider that it is not possible to assess the influence of S19W common variants in Q97X heterozygous subjects, given the limited number of subjects in this family.

Regarding Apo C-III levels, both the mother (subject 7) and one of the affected sisters of the proband (subject 12) in our study showed elevated levels of Apo C-III. It is remarkable that both were the only ones displaying severe HTG (>5.6 mmol/L) at the time the test was performed in 2006, when even the proband (who was under treatment) showed normal TG levels and also normal levels of this apolipoprotein (Figure 1). In previous reports on Q97X and other APOA5 mutations that lead to Apo A-V deficiency, higher levels of Apo C-III in the mutation carriers have also been described [48,49,52,53]. In fact, a 2.7-fold increase of Apo C-III was observed in Apo A-V deficient plasma compared to control plasma [48]. Furthermore, the S19W and −1131 T>C variants in the APOA5 gene have been associated with higher levels of Apo C-III [60]. On the other hand, it has also been described that hypertriglyceridemic patients display higher levels of this apolipoprotein compared to controls [61]. All these observations suggest a complex relation between Apo A-V and Apo C-III. Therefore, it is possible that the impaired TG hydrolysis in Apo A-V deficient plasma is a consequence of the lack of an LPL activator (Apo A-V), as well as the presence of an inhibitor (Apo C-III) [62]. The proband in our study, who is homozygous for the Q97X mutation and should have a deficiency of Apo A-V in plasma, did not show elevated levels of Apo C-III. Therefore, the observation of normal Apo C-III in the proband may suggest that, in our patients, plasma Apo C-III levels are mainly determined by HTG itself and not by the Apo A-V deficiency.

## Conclusions

We have identified a mutation in the APOA5 gene (p.Gln97Ter; Q97X) responsible for severe HTG in a consanguineous Chilean family using a genome-wide approach based on the homozygosity mapping strategy. The causative locus was initially located in the APOA5/A4/C3/A1 cluster and the mutation was finally found in the APOA5 gene as a nonsense substitution Q97X. In our family, homozygotes for Q97X showed plasma TG levels > 112 mmol/L, while heterozygous family members showed a less severe phenotype. In general, the phenotypic expression of this mutation is highly variable, especially in heterozygous, where adverse environmental factors and other pathologic conditions may play a fundamental role in the manifestation of the disease.

## Abbreviations

Apo: Apolipoprotein; Apo AV: Apolipoprotein AV; GWAS: Genome Wide Association Studies; HLP: Hyperlipoproteinemia; HTG: Hypertriglyceridemia; LPL: Lipoprotein Lipase; PCR: Polymerase Chain Reaction; PUFA: Polyunsaturated fatty acid; QM: Chylomicron; RFLP: Restriction Fragment Length Polymorphism; SNP: Single Nucleotide Polymorphism; TG: Triglyceride; VLDL: Very Low Density Lipoprotein.

## Competing interests

The authors declare that they have no competing interests.

## Authors’ contributions

CD: AB, ES, FG VS: ES, FG AM: ES, FG SE: ES LRC: AB MC: ES SV S: AB MF: AB AR: AB LR: AB CF L: AB JAM: FG JLS: AB, ES, FG. All authors read and approved the final manuscript.

## Pre-publication history

The pre-publication history for this paper can be accessed here:

http://www.biomedcentral.com/1471-2350/13/106/prepub

## Supplementary Material

Additional file 1 **Supplemental data.** APOA5 Q97X mutation identified through homozygosity mapping causes severe hypertriglyceridemia in a consanguineous family. **Supplemental tables: Table S1**. Genes related to hypertriglyceridemia. **Table S2**. Features of the human Cyto SNP-12 panel (as reported by Illumina http://www.illumina.com). **Table S3.** Regions showing excess of homozygosity in the two affected sisters (family members 11 and 12) and more than 20% of heterozygosis in a healthy sister (subject 14). **Table S4**. Main clinical features of the 97X homozygous cases reported of Q97X mutation in APOA5. Supplemental figures: **Figure S1**. Pedigree chart of the complete family. **Figure S2**. PCR-RFLP analysis of S19W and -1131 T>C polymorphisms of the APOA5 gene in the complete consanguineous Chilean family with HTG and APOA5 mutation. **Figure S3.** Single nucleotide substitution in exon 4 (c.289 C>T), which converts the glutamine codon at position 97 into a termination codon (Q97X) in APOA5 gene. **Figure S4.** Model of Apo A-V protein structure and critical domains. Click here for file
