# Supplementary material for: APOA5 Q97X Mutation Identified through homozygosity mapping causes severe hypertriglyceridemia in a Chilean consanguineous family
Source: BMC Med Genet. 2012 Nov 15;13:106. doi: 10.1186/1471-2350-13-106 (PMC3523038; doi:10.1186/1471-2350-13-106)
Supplement: Additional file 1 — Supplemental data. APOA5 Q97X mutation identified through homozygosity mapping causes severe hypertriglyceridemia in a consanguineous family. Supplemental tables: Table S1. Genes related to hypertriglyceridemia. Table S2. Features of the human Cyto SNP-12 panel (as reported by Illumina http://www.illumina.com). Table S3. Regions showing excess of homozygosity in the two affected sisters (family members 11 and 12) and more than 20% of heterozygosis in a healthy sister (subject 14). Table S4. Main clinical features of the 97X homozygous cases reported of Q97X mutation in APOA5. Supplemental figures: Figure S1. Pedigree chart of the complete family. Figure S2. PCR-RFLP analysis of S19W and -1131 T>C polymorphisms of the APOA5 gene in the complete consanguineous Chilean family with HTG and APOA5 mutation. Figure S3. Single nucleotide substitution in exon 4 (c.289 C>T), which converts the glutamine codon at position 97 into a termination codon (Q97X) in APOA5 gene. Figure S4. Model of Apo A-V protein structure and critical domains. [file 1471-2350-13-106-S1.docx]

**SUPPLEMENTAL DATA**

**APOA5 Q97X MUTATION IDENTIFIED THROUGH HOMOZYGOSITY MAPPING CAUSES SEVERE HYPERTRIGLYCERIDEMIA IN A CONSANGUINEOUS FAMILY**

**APOA5 Sequencing and PCR- RFLP analysis**

The coding sequences of exon 2 and 3 were amplified using the primers APOA5gF1 (5'-CAGGTGGGCAGGGGAGAGGT GGTA-3') and APOA5sR2 (5'-CCAGCAGCGGCCACAGAGGTTGAG-3'). Exon 4 was divided into two regions, and previously reported primers were used^1^ (Oliva et al. 2005). For the 5' half of exon 4, the primers used were 4F (5'-CGGCCTGGATATCTGTC CCC-3') and 4th (5'-CCCAGTG CCTGCAAAGGCTC-3 ') with an amplification product of 662 bp. For the 3' half of exon 4, the primers used were 4EF (5'-GGTGCTCTCCCGGAAGC TCA-3') and 4R (5'-GCCTCTCCCTCCCTACTCCC-3 ') with an amplification product of 707 bp. The amplification conditions for exons 2 and 3 were 94°C for 5 minutes, 94°C for 30 seconds, 62°C for 35 seconds and 72°C for 50 seconds for 25 cycles, followed by 7 minutes at 72°C. The amplification conditions for the 5' half of exon 4 were 94°C for 15 minutes (Hot Start), 94°C for 40 seconds, 55°C for 40 seconds, 72°C for 80 seconds for 30 cycles, followed by 7 minutes at 72°C. For the 3' half of exon 4, the amplification conditions were the same as for the 5' region, except for the annealing temperature which was increased to 61°C.

The homozygous mutation found by sequencing (c.289C> T), which eliminates a PstI restriction site, was confirmed by a restriction fragment analysis (PCR-RFLP) of PCR – amplified 5’ region of exon 4. The primers used were 4F (5’-CGGCCTGGATATCTGTCCCC-3’) and 4eR (5’-CCCAG TGCCTGCAAAGGCTC-3’). In the Wild-Type sequence (WT) 289C/C, five restriction fragments of 265, 157, 153, 78 and 9 bp were obtained. In the mutant homozygous sequence 289T / T only four restriction fragments of 310, 265, 78 and 9 bp were obtained. The amplification product (662 bp) was digested with PstI (Fast Digest, Fermentas) for 30 minutes at 37°C and the fragments were separated in 3.5% agarose gel electrophoresis.

**Genotype determination of S19W and -1131T>C polymorphisms in the APOA5 gene**

For S19W, the following PCR primers were used: S19WF (5’GGCTCTTCT TTCAGGTGGGTCTCCG-3’) and S19WR (3’-GCCTTTCCGTGCCTGGGTGG T-5’). The amplification conditions were 95ºC for 5 minutes, 95ºC for 1 minute, 62ºC for 1 minute, 72ºC for 1 minute for 30 cycles, followed by 10 minutes at 72ºC. The amplification product (157 bp) was digested with Taq 1 and the fragments (157 bp for the wild type and 134 and 23 bp for the mutant allele) were separated in a 4% agarose gel electrophoresis.

For -1131T>C, the following PCR primers were used: -1131T>CF (5’-GG AGCTTGTGAACGTGTGTATGAGT-3’) and -1131T>CR (3’-CCCAGGAACTG GAGCGAAATT-5’). The amplification conditions were 95ºC for 5 minutes, 95ºC for 1 minute, 58ºC for one minute for 30 cycles, followed by 72ºC for 7 minutes. The amplification product (154 bp) was digested with Tru1L and the fragments (133 and 21 bp for the wild type and 154 bp for the mutated allele) were separated in 4% agarose gel electrophoresis.

**Molecular modeling of Apo A-V protein structure**

The amino acid sequence of human ApoA5 (366 residues) was retrieved from Uniprot (accession entry Q6Q788).^2^ Using the Protein Data Bank ([www.pdb.org](http://www.pdb.org)) sequence search tool,^3^ the crystal structure of lipid-free human apolipoprotein A-I was identified as a suitable template for modeling (PDB id 2A01, resolution 2,4Å).^4^ PFAM search also identify Apo A-V as part of apolipoprotein (PF01442) family, which also includes the Apo A-I, Apo A-IV and Apo E proteins, further supporting the selection of Apo A-I as target for modeling.^5^ Apo A-V is predicted to have a signal peptide with a cleavage site probability of 0.895 between residues 23 and 24 according to the SignalP3.0 server.^6^ Secondary structure predictions were performed using the PCI-SS server.^7^ After several rounds of optimization, a feasible alignment to construct a 3D model for ApoA5 residues 1-278 was achieved. The remaining 88 residues, corresponding to the C-terminal segment, were not included. The final alignment is shown in figure 1, presenting 18.4 and 44.8% of sequence identity and sequence similarity between the template structure and ApoA5, respectively. Comparative modeling was performed using MODELLER ^8^ as implemented in the Build Homology Models protocol in Discovery Studio v2.1 (Accelrys Inc., San Diego, USA). Fifty models were produced and the top scoring, as ranked by the program DOPE score, was selected. Model quality assessment was performed using the RAMPAGE server and Verify 3D profiles.^9,10^


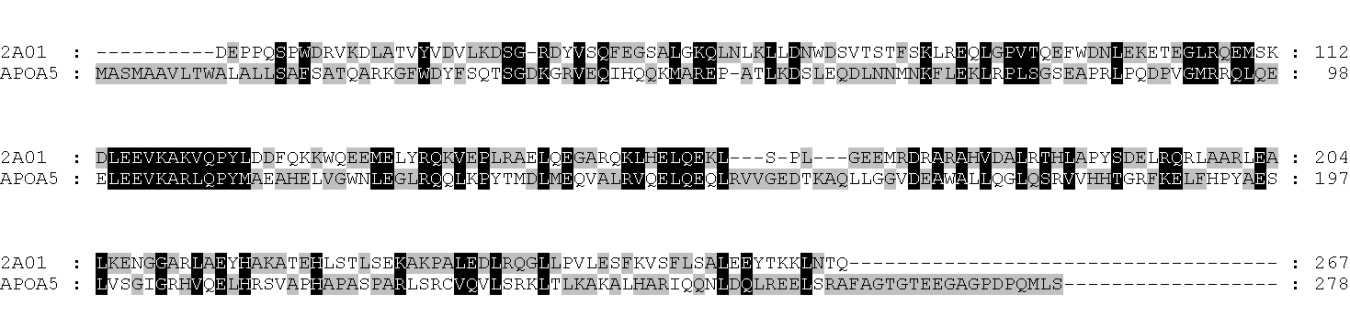


Multiple amino acid sequence alignment of Apo A-I with modeled Apo A-V obtained by using CLUSTALX 2.0 and visualized using GeneDoc.^11,12^

**LOD score calculation: recessive model of inheritance**

This page shows the output of the program Linkage 5.1 for the haplotype flanking the APOA5 gene rs3741301-rs10488698-rs664059-rs1263173-rs2849176-rs11216162 (Figure 1). We used the following genetic model: recessive inheritance with complete penetrance and absence of phenocopies, a disease causing allele frequency of 0.0001, and haplotype frequencies of 0.01 (disease-linked haplotype: AGGAGG), 0.05, 0.05, 0.05, 0.05, and 0.79 (all other possible haplotypes). The maximum LOD score was 2.01 at theta = 0.

LINKAGE (V5.1) WITH 2-POINT AUTOSOMAL DATA

ORDER OF LOCI: 1 2

-----------------------------------

THETAS 0.500

-----------------------------------

PEDIGREE | LN LIKE | LOG 10 LIKE

-----------------------------------

1 -42.368985 -18.400577

-----------------------------------

TOTALS -42.368985 -18.400577

-2 LN(LIKE) = 8.47379691175678E+0001 LOD SCORE = 0.000000

-----------------------------------

-----------------------------------

THETAS 0.000

-----------------------------------

PEDIGREE | LN LIKE | LOG 10 LIKE

-----------------------------------

1 -37.728315 -16.385164

-----------------------------------

TOTALS -37.728315 -16.385164

-2 LN(LIKE) = 7.54566291730162E+0001 LOD SCORE = 2.015413

-----------------------------------

-----------------------------------

THETAS 0.100

-----------------------------------

PEDIGREE | LN LIKE | LOG 10 LIKE

-----------------------------------

1 -38.568224 -16.749931

-----------------------------------

TOTALS -38.568224 -16.749931

-2 LN(LIKE) = 7.71364480662707E+0001 LOD SCORE = 1.650646

-----------------------------------

-----------------------------------

THETAS 0.200

-----------------------------------

PEDIGREE | LN LIKE | LOG 10 LIKE

-----------------------------------

1 -39.480695 -17.146211

-----------------------------------

TOTALS -39.480695 -17.146211

-2 LN(LIKE) = 7.89613901147158E+0001 LOD SCORE = 1.254366

-----------------------------------

-----------------------------------

THETAS 0.300

-----------------------------------

PEDIGREE | LN LIKE | LOG 10 LIKE

-----------------------------------

1 -40.436372 -17.561256

-----------------------------------

TOTALS -40.436372 -17.561256

-2 LN(LIKE) = 8.08727439697501E+0001 LOD SCORE = 0.839321

-----------------------------------

-----------------------------------

THETAS 0.400

-----------------------------------

PEDIGREE | LN LIKE | LOG 10 LIKE

-----------------------------------

1 -41.402899 -17.981012

-----------------------------------

TOTALS -41.402899 -17.981012

-2 LN(LIKE) = 8.28057984655827E+0001 LOD SCORE = 0.419565

**LOD score calculation: dominant model of inheritance**

This page shows the output of the program Linkage 5.1 for the haplotype flanking the APOA5 gene rs3741301-rs10488698-rs664059-rs1263173-rs2849176-rs11216162 (Figure 1). We used the following genetic model: dominant inheritance with complete penetrance and absence of phenocopies, a disease causing allele frequency of 0.0001, and haplotype frequencies of 0.01 (disease-linked haplotype: AGGAGG), 0.05, 0.05, 0.05, 0.05, and 0.79 (all other possible haplotypes). The maximum LOD score was 2.17 at theta = 0.

LINKAGE (V5.1) WITH 2-POINT AUTOSOMAL DATA

ORDER OF LOCI: 1 2

-----------------------------------

THETAS 0.500

-----------------------------------

PEDIGREE | LN LIKE | LOG 10 LIKE

-----------------------------------

1 -43.321925 -18.814433

-----------------------------------

TOTALS -43.321925 -18.814433

-2 LN(LIKE) = 8.66438492464930E+0001 LOD SCORE = 0.000000

-----------------------------------

-----------------------------------

THETAS 0.000

-----------------------------------

PEDIGREE | LN LIKE | LOG 10 LIKE

-----------------------------------

1 -38.316413 -16.640571

-----------------------------------

TOTALS -38.316413 -16.640571

-2 LN(LIKE) = 7.66328268438353E+0001 LOD SCORE = 2.173861

-----------------------------------

-----------------------------------

THETAS 0.100

-----------------------------------

PEDIGREE | LN LIKE | LOG 10 LIKE

-----------------------------------

1 -39.265380 -17.052701

-----------------------------------

TOTALS -39.265380 -17.052701

-2 LN(LIKE) = 7.85307590116400E+0001 LOD SCORE = 1.761731

-----------------------------------

-----------------------------------

THETAS 0.200

-----------------------------------

PEDIGREE | LN LIKE | LOG 10 LIKE

-----------------------------------

1 -40.305830 -17.504562

-----------------------------------

TOTALS -40.305830 -17.504562

-2 LN(LIKE) = 8.06116603330412E+0001 LOD SCORE = 1.309870

-----------------------------------

-----------------------------------

THETAS 0.300

-----------------------------------

PEDIGREE | LN LIKE | LOG 10 LIKE

-----------------------------------

1 -41.392761 -17.976609

-----------------------------------

TOTALS -41.392761 -17.976609

-2 LN(LIKE) = 8.27855213815487E+0001 LOD SCORE = 0.837823

-----------------------------------

-----------------------------------

THETAS 0.400

-----------------------------------

PEDIGREE | LN LIKE | LOG 10 LIKE

-----------------------------------

1 -42.430370 -18.427236

-----------------------------------

TOTALS -42.430370 -18.427236

-2 LN(LIKE) = 8.48607391754137E+0001 LOD SCORE = 0.387197

**SUPPLEMENTAL REFERENCES**

1. Oliva CP, Pisciotta L, Li Volti G, Sambataro MP, Cantafora A, Bellocchio [Catapano A](http://www.ncbi.nlm.nih.gov/pubmed?term=%22Catapano%20A%22%5BAuthor%5D), [Tarugi P](http://www.ncbi.nlm.nih.gov/pubmed?term=%22Tarugi%20P%22%5BAuthor%5D), [Bertolini S](http://www.ncbi.nlm.nih.gov/pubmed?term=%22Bertolini%20S%22%5BAuthor%5D), [Calandra S](http://www.ncbi.nlm.nih.gov/pubmed?term=%22Calandra%20S%22%5BAuthor%5D): **Inherited apolipoprotein A-V deficiency in severe hypertriglyceridemia**. *Arterioscler Thromb Vasc Biol* 2005, 25:411-417
2. Consortium TU 2010: **The universal protein resource (uniprot) in 2010.** *Nucleic Acids Res* 2010*,*  38:D142-D148
3. Berman HM, Westbrook J, Feng Z, Gilliland G, Bhat TN, Weissig H, Shindyalov IN, Bourne PE: **The protein data bank.** *Nucleic Acids Res* 2000, 28:235-242
4. Ajees AA, Anantharamaiah GM, Mishra VK, Hussain MM, Murty HM: **Crystal structure of human apolipoprotein a-1: Insights into its protective effect against cardiovascular disease**. *Proc Natl Acad Sci U S* *A* 2006, 103:2126-2131.
5. Finn RD, Mistry J, Tate J, Coggil P, Heger A, Pollington JE, Gavin OL, Gunasekaran P, Ceric G, Forslund K, Holm L, Sonnhammer ELL, Eddy SR, Bateman A; **The pfam protein families database.** *Nucleic Acids Res* 2010, 38:D211-222
6. Emanuelsson O, Brunak S, Von Heijne G, Nielsen H: **Locating proteins in the cell using target signal and related tools**. *Nat Protoc* 2007, 2:953-971
7. Green J, Korenberg M, Aboul-Magd M: **Pci-ss: Miso dynamic nonlinear protein secondary structure prediction**. *BMC Bioinformatics* 2009, 10:222
8. Sali A: **Comparative protein modeling by satisfaction of spatial restraints***, Mol Med Today* 1995, 1:270-277
9. Eisenberg D, Luthy R, Bowie JU: **Verfy3d: Assessment of protein models with three-dimensional profiles**. [*Methods Enzymol*](javascript:AL_get(this,%20'jour',%20'Methods%20Enzymol.');) 1997, 277:396-404.
10. Lovell SC, Davis IW, Arendall WB, de Bakker PI, Word JM, Prisant MG, Richardson JS, Richardson DC: **Structure validation by c-alpha geometry: Phi psi and c-beta deviation**. *Proteins* 2003, 50:437-450
11. Larkin MA, Blackshields G, Brown NP, Chenna R, McGettigan PA, Mc William H, Valentin F, Wallace IM, Wilm A, Lopez R, Thompson JD, Gibson TJ, Higgins DG: **Clustal w and clustal x version 2.0.** *Bioinformatics* 2007, 23:2947-2948
12. Nicholas KB, HBN, Deerfield DW Genedoc: **Analysis and visualization of genetic variation**. *EMBNEWS.NEWS* 1997, 4:14

**SUPPLEMENTAL TABLES**

**Table S1**

Genes related to hypertriglyceridemia

| **Gene** | **Protein** | **Chromosomal location** |
| --- | --- | --- |
| ANGPTL3 | Angiopoietin-like 3 | 1p31.1-p22.3 |
| APOA5 | Apolipoprotein AV | 11q23 |
| APOB | Apolipoprotein B | 2p24-p23 |
| APOC2 | Apolipoprotein CII | 19q13,2 |
| APOC3 | Apolipoprotein CIII | 11q23.1-q23.2 |
| APOE | Apolipoprotein E | 19q13.2 |
| CHREBP | Carbohydrate response element binding protein | 7q11.23 |
| GALNT2 | UDP-N-acetyl-alpha-D-galactosamine:polypeptide N-acetylgalactosaminyltransferase 2 | 1q41-q42 |
| GCKR | Glucokinase regulatory protein | 2p23 |
| GPIHBP1 | Glycosylphosphatidylinositol anchored high density lipoprotein binding protein 1 | 8q24.3 |
| LIPC | Lipase, hepatic | 15q21-q23 |
| LMF1 | Lipase maturation factor 1 | 8p22 |
| NCAN | Neurocan | 19p12 |
| TRIB1 | Tribbles homolog 1 (Drosophila) | 8q24.13 |

**Table S2**

Features of the human Cyto SNP-12 panel (as reported by Illumina http://www.illumina.com)

| Number of markers per sample | 299,140 |
| --- | --- |
| Genomic coverage (mean) |  |
| CEU | 0.81 |
| CHB + JPT | 0.83 |
| YRI | 0.55 |
| Minor allele frequency (mean) |  |
| CEU | 0.22 |
| CHB + JPT | 0.21 |
| YRI | 0.21 |
| Spacing (mean, kb) | 9.7 |
| Call frequency | > 99% |
| Reproducibility | > 99.9% |
| Mendelian inconsistencies | < 0.1% |
| Hapmap concordance | 99.7% |

**Legend to Table S2:** CEU: Utah residents with Northern and Western European ancestry from the CEPH collection, CHB: Han Chinese in Beijing, China, JPT: Japanese in Tokyo, Japan, YRI: Yoruban in Ibadan, Nigeria.

**Table S3**

Regions showing excess of homozygosity in the two affected sisters (family members 11 and 12) and more than 20% of heterozygosis in a healthy sister (subject 14)

|  |  |  |  |  |  |
| --- | --- | --- | --- | --- | --- |
| **Chr** | **Begins (nt.)** | **Ends (nt.)** | **Size (nt.)** | **Nº of SNP** | **Heterozygosity in non-affected sister (%)** |
| 2 | 98649780 | 99699203 | 1049423 | 141 | 17 |
| 2 | 196122832 | 197739165 | 1616333 | 146 | 13 |
| 5 | 111730450 | 112620505 | 890055 | 129 | 10.9 |
| 6 | 80611759 | 82144016 | 1532257 | 104 | 41.3 |
| 7 | 48877226 | 49595753 | 718527 | 108 | 21.3 |
| 7 | 53635572 | 54458878 | 823306 | 131 | 12.2 |
| 7 | 68382440 | 69510315 | 1127875 | 125 | 39.2 |
| 11 | 23675319 | 51369372 | 27694053 | 2896 | 28.7 |
| 11 | 51386817 | 58203184 | 6816367 | 349 | 26.4 |
| 11 | 58216649 | 58977455 | 760806 | 108 | 24.1 |
| 11 | 89688030 | 121320701 | 31632671 | 2965 | 33.6 |
| 14 | 91940038 | 93293352 | 1353314 | 202 | 25.7 |
| 16 | 46545109 | 47430237 | 885128 | 132 | 20.5 |
| 19 | 23288119 | 23553353 | 265234 | 116 | 36.2 |
| 19 | 33435046 | 33619283 | 184237 | 168 | 29.2 |

**Legend to Table S3:** Chr: chromosome; nt: nucleotides

**Table S4**

Main clinical features of the 97X homozygous cases reported of Q97X mutation in APOA5

|  | **Oliva et al. (2008)** | **Charriere et al. (2009)** | **Dussaillant et al. (herein)** | **Dussaillant et al. (herein)** |
| --- | --- | --- | --- | --- |
| **Sex** | M | M | F | F |
| **Age (years)** | 17 | 58 | 46 | 50 |
| **Age at diagnosis (years)** | 2 | 25 | 22 | 30 |
| **Maximum TG (mmol/L)** | 11 | 40 | 112 | 116 |
| **HDL (mmol/L)** | 0.6 | 0.5 | 0.8 | 0.5 |
| **BMI (Kg/m^2^)** | 19 | 21 | 23 | 28 |
| **Other pathologies** | No | No | No | Prediabetes |
| **Clinical manifestations** | Xantomatosis | No pancreatitis | Pancreatitis | Pancreatitis |
| **Plasma Apo A-V** | Not detectable | Not detectable | Not evaluated | Not evaluated |
| **Treatment** | Diet and w3 fatty acids | Not described | Diet, fibrates, and nicotinic acid | Diet, fibrates and w3 fatty acids |

**Legend to Table S4:** TG: Triglycerides; HDL: High Density Lipoprotein; BMI: Body Mass Index; M: Male; F: Female

**SUPPLEMENTAL FIGURES**

**Figure S1**

Pedigree chart of the complete family


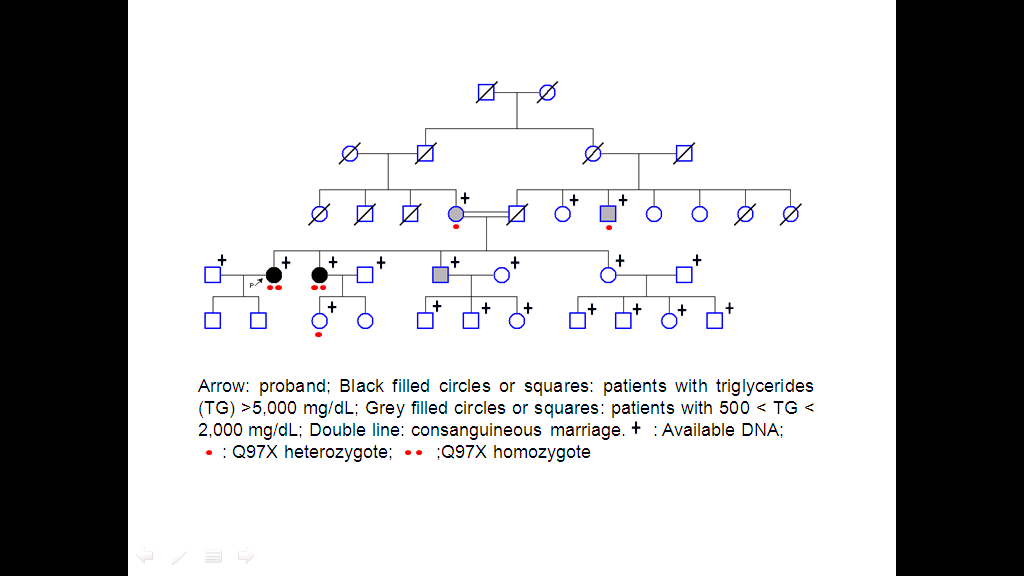


The daughter of the affected sister of the proband with available DNA was identified by DNA analysis as a carrier of the Q97X mutation in heterozygous state. At the moment of the study, she was 17 years-old, with an IMC of 25.3 kg/m2, no signs of diabetes or prediabetes, plasma TG of 2 mmol/L, plasma Apo-CIII of 16.8 mg/dL and plasma Apo-CIII of 6.6 mg/dL. This subject never developed lipid disorders, probably due to her young age and the lack of other comorbid conditions which often triggers HTG in susceptible individuals. In total, there are four obligate carriers of the Q97X mutation in the youngest generation (all descendants of the proband and her affected sister).

**Figure S2**

PCR-RFLP analysis of S19W and -1131 T>C polymorphisms of the APOA5 gene in the complete consanguineous Chilean family with HTG and APOA5 mutation

| **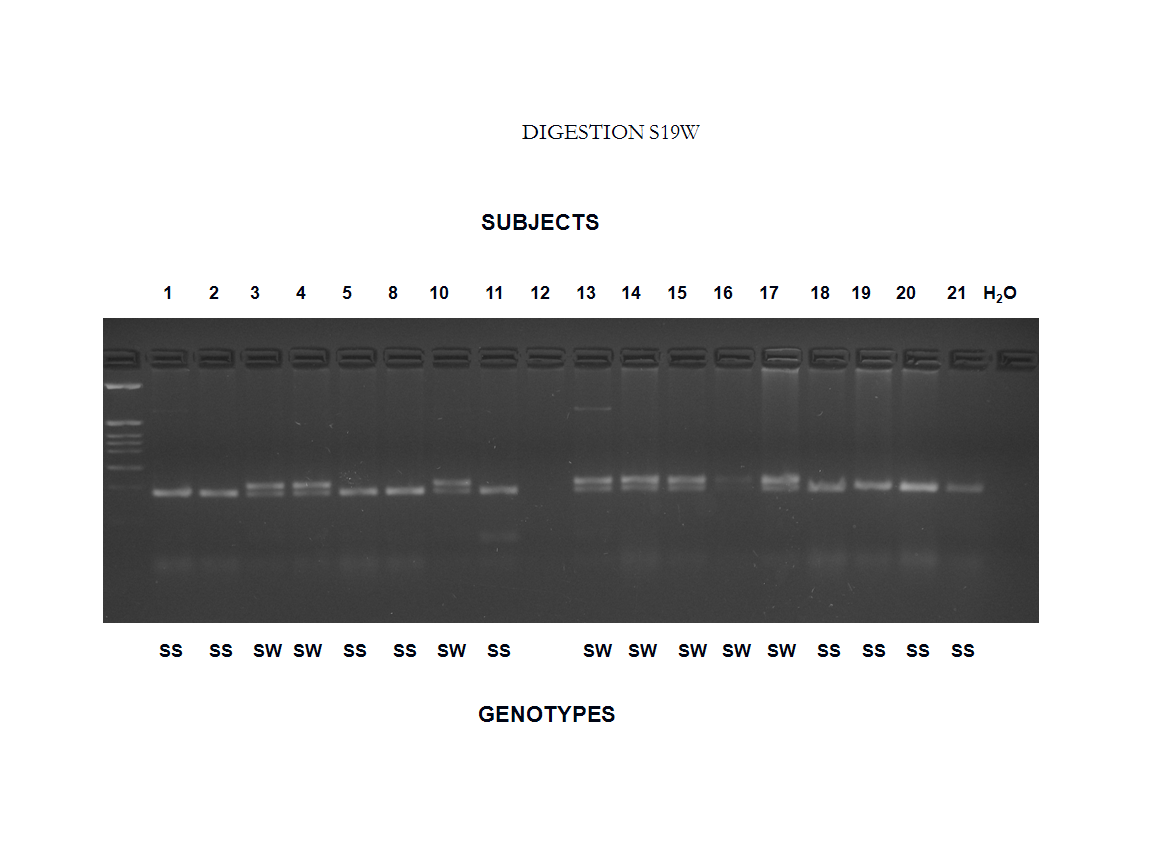** | **PCR-RFLP S19W** |
| --- | --- |
| **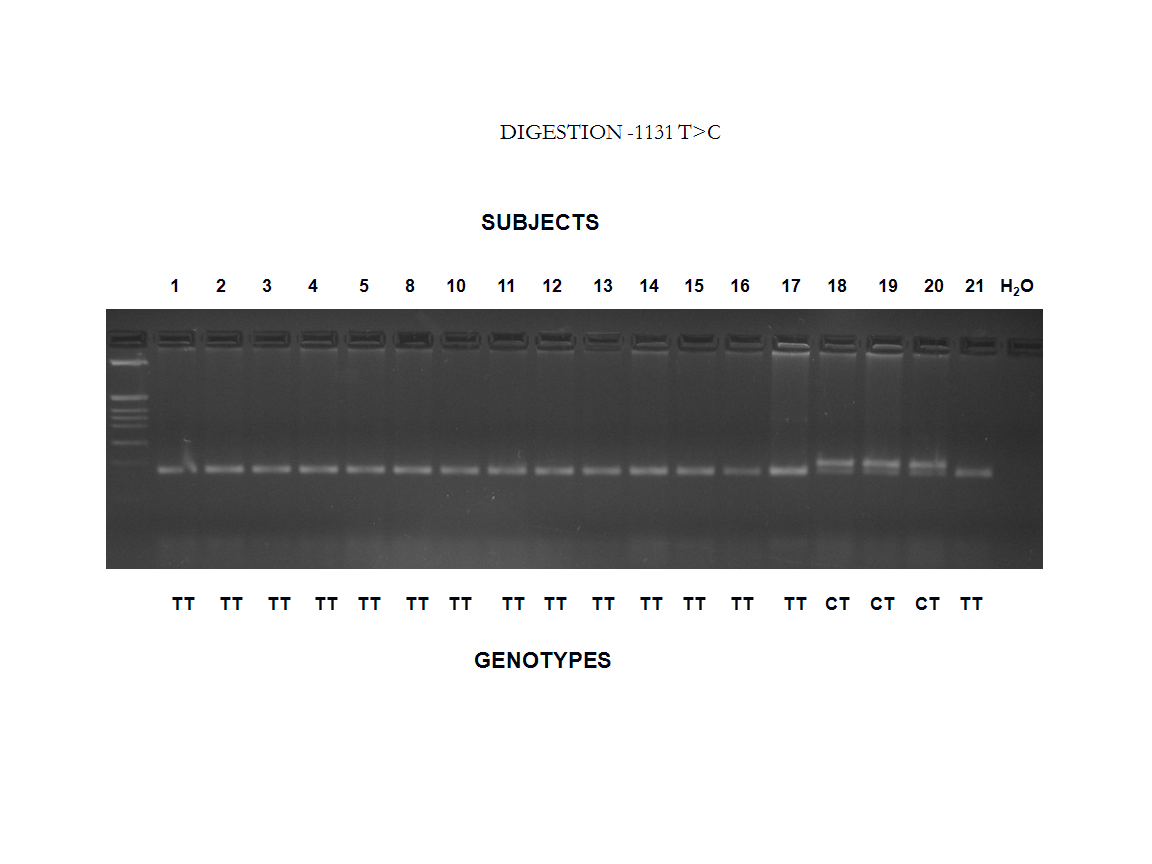** | **PCR-RFLP**  **-1131 T>C** |

**Legend to figure S2:** 1 and 2: affected sisters (Q97X homozygotes); 3 and 4: unaffected siblings; 5: mother of the affected sisters (Q97X heterozygote); 8: daughter of affected sister (Q97X heterozygote); 16: paternal uncle of the affected sisters (Q97X heterozygote); 10-15, 17-21: other studied family members.

**Figure S3**

Single nucleotide substitution in exon 4 (c.289 C>T), which converts the glutamine codon at position 97 into a termination codon (Q97X) in APOA5 gene

| 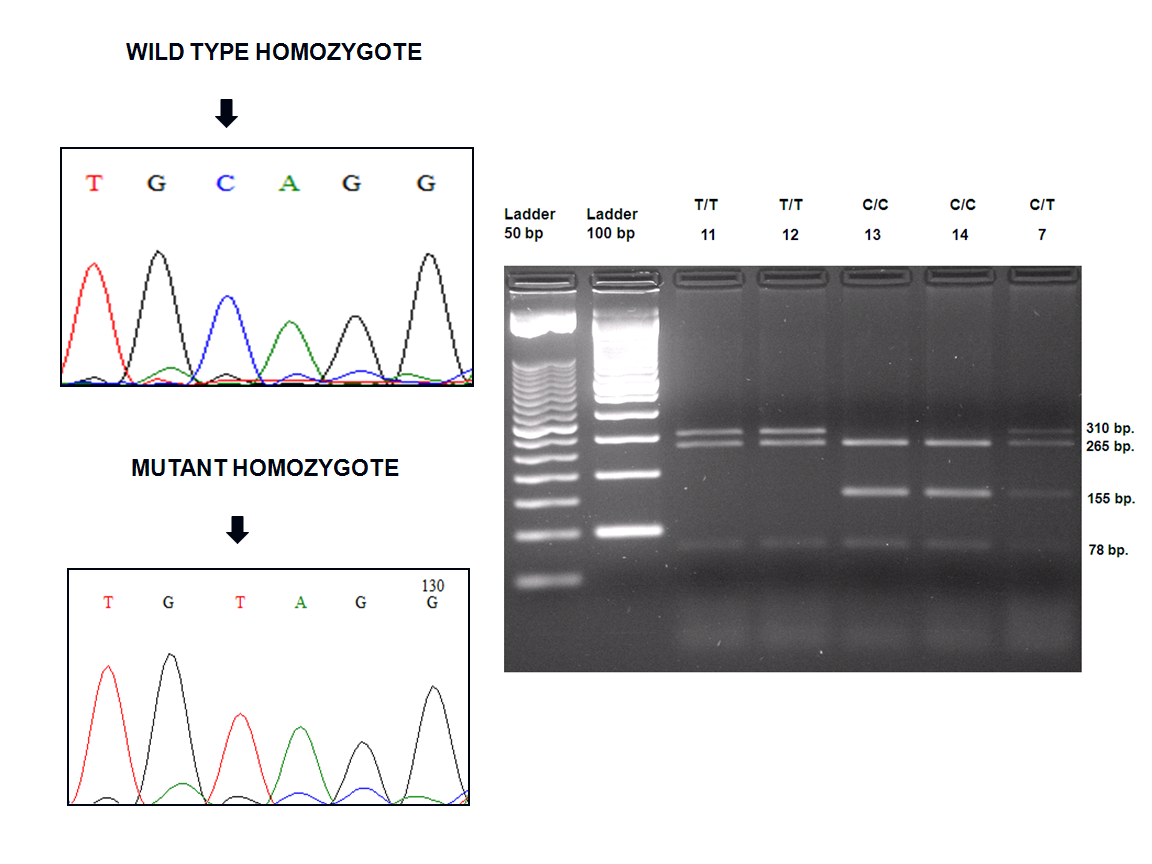 | 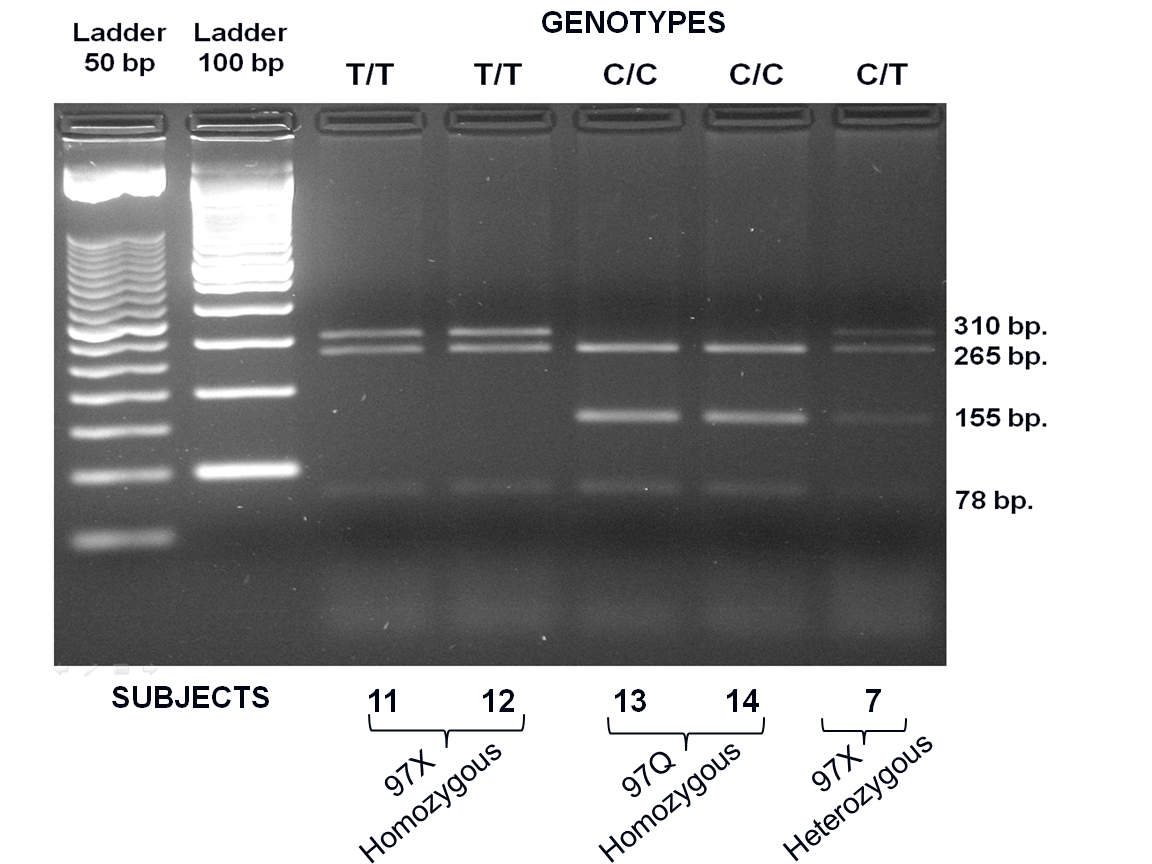 |
| --- | --- |

**Legend to figure S3:** 11: proband; 12: affected sister; 13 and 14: non-affected siblings; 7: mother (Intermediate phenotype). The proband and her sister are homozygous for a C>T substitution. The mother is heterozygous for the same mutation.

**Figure S 4**

Model of Apo A-V protein structure and critical domains

**
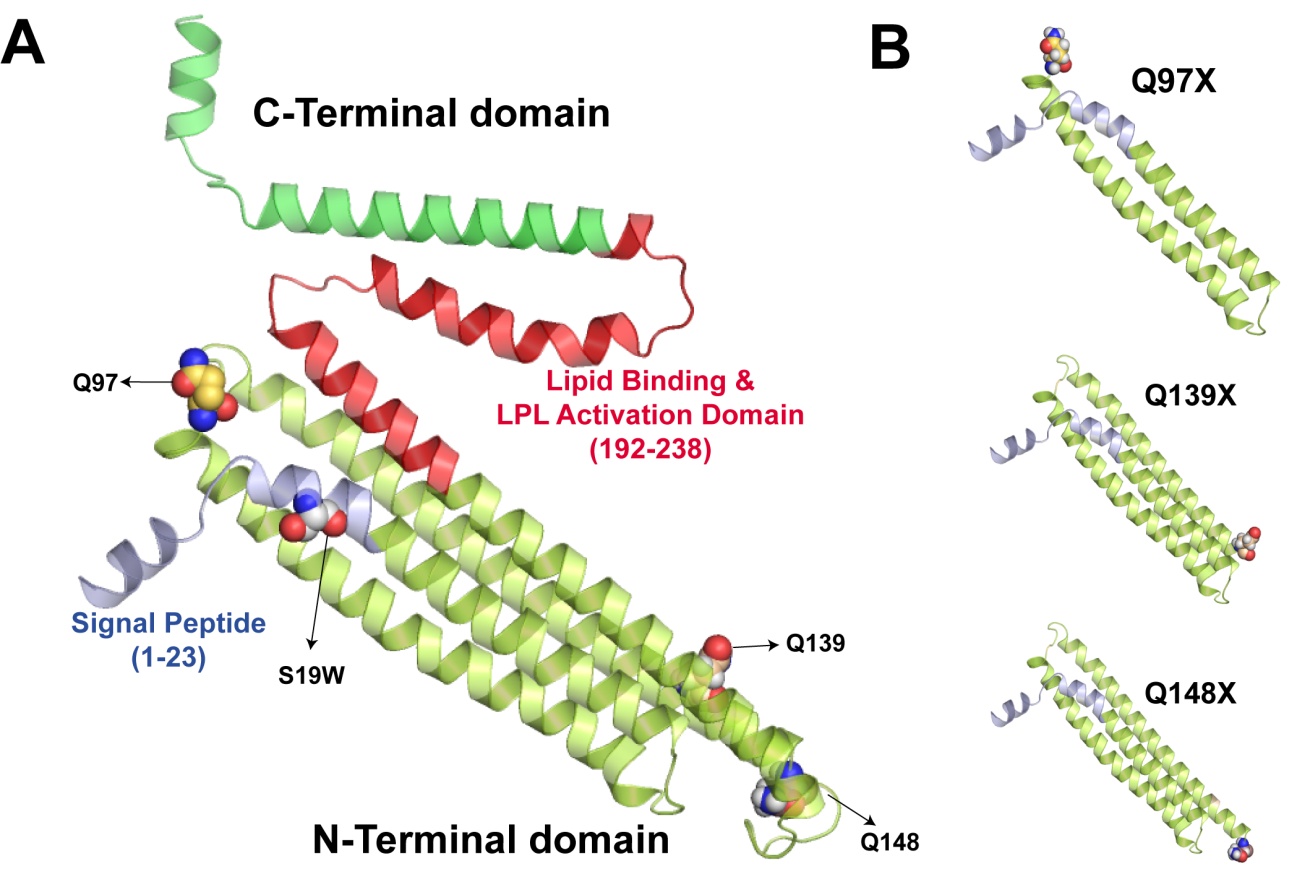
**

**Legend to Figure S4:**

**A:** Predicted model of Apo A-V with its most important domains (For detailed information see supplemental methods). The picture shows Q97X and other previously described mutations that severely affect the protein structure (Q139X and Q148X).

In blue: signal peptide; in yellow: N-terminal domain, important for lipid binding, in red: lipid binding and LPL activation domain; in green: C-terminal domain, important for lipid binding (Wong and O’ Ryan 2007, Wong et al 2009)

**B:** Predicted truncated peptides resulting from Q97X, Q139X and Q148X mutations. If synthesized, they all lack the lipid binding and LPL activation domain, although they still conserve a portion of the lipid-binding domain (N-terminal domain)
